# More prevalent and more severe: gender differences of depressive symptoms in Chinese adolescents

**DOI:** 10.3389/fpubh.2023.1167234

**Published:** 2023-07-13

**Authors:** Yue Sun, Yiwen Zhong, Wenzhao Sun, Lingjun Chu, Jiang Long, Xi Wang Fan

**Affiliations:** ^1^Psychological Assessment and Research Center, Clinical Research Center for Mental Disorders, Shanghai Pudong New Area Mental Health Center, School of Medicine, Tongji University, Shanghai, China; ^2^Department of Internal Medicine, Yale School of Medicine, Yale University, New Haven, CT, United States; ^3^The Centre of Excellence for Research in AIDS (CERiA), Faculty of Medicine, University of Malaya, Kuala Lumpur, Malaysia

**Keywords:** depression, gender difference, adolscent, major depressive disorder, depressed adolescents

## Abstract

**Background:**

Adolescent depression has become a leading problem around the world, especially as the COVID-19 pandemic has remained prevalent and heavily influenced people’s mental health. While gender difference has always been a topic in the field of psychiatry, there are cultural differences across the world that must be taken into account. The current study is examining gender differences in symptoms of Chinese adolescents with depression.

**Methods:**

The sample was obtained from a total of 574 adolescent patients (172 males and 402 females) diagnosed with depression following the DSM-IV/ICD-10 diagnostic criteria; patients who also had other severe mental or physical illnesses were excluded. The ages of participants ranged from 10 to 19 years. Additionally, independent *t*-test and one-way ANOVA were used to examine differences in symptoms between different gender and age groups. The LPA was used to examine whether females and males were having different patterns of symptoms.

**Results:**

Our analysis showed that compared to males, females exhibited higher rates of depression and more severe depressive symptoms across age groups. Likewise, the analysis also revealed an earlier onset of depression among Chinese adolescents compared to that in Western countries in previous studies. Finally, the LPA showed that mild to moderate depression was predominant in male patients, while severe depression was predominant in female patients.

**Conclusion:**

This study highlights the gender differences in the prevalence and severity of depressive symptoms in Chinese adolescents. The current study highlighted the importance of gender equality and developing gender-friendly interventions in maintaining the overall mental health of adolescents in China.

## 1. Introduction

Depression is a complex and chronic mental illness that usually develops in childhood or adolescence and affects the overall quality of life for patients later in life. Moreover, adolescent depression has become a far more common mental health problem. An estimated 24.6% of Chinese teenagers suffer from depression, with 7.4% suffering from severe depression (1). A recent meta-analysis estimated that there was 17.2% of children and adolescence (6–15 years) in China reported depressive symptoms (2).

Notable differences were observed between genders in terms of both the prevalence and clinical symptoms of depression, first highlighted the variance in depression by noting that women were approximately twice as likely as men to suffer from depression (3). Moreover, the existence of this difference is not unique to adults: although several past studies have shown that there is no gender difference in the prevalence of depression in boys and girls throughout childhood (4, 5), the finding that apparent gender differences in depression between males and females emerge in early adolescence and widen thereafter between the ages of 15–18 years (4, 6, 7), is now widely accepted.

Much of this general consensus is based on studies conducted with participants from Western countries, particularly from the United States. However, a WHO study that collected data from 18 countries indicated that gender differences in the incidence of depression do vary widely across countries (8, 9). When examining depressive symptoms in detail, a meta-analysis of cross-sectional studies of Chinese children and adolescents showed that the prevalence of depressive symptoms in Chinese children and adolescents was slightly higher for boys (16.8%) than for girls (15.6%) (10, 11), suggesting that gender differences in terms of depressive symptoms among Chinese adolescents may differ from the figures found in previous studies.

## 2. Materials and methods

### 2.1. Participants

The sample used in this study was obtained from a total of 574 patients (172 males and 402 females) diagnosed with adolescent depression by specialists in the outpatient clinic of the Mental Health Center affiliated with Tongji University in Shanghai, China from March 2021 to March 2022. The diagnosis of adolescent depression followed the DSM-IV/ICD-10 diagnostic criteria; patients with other severe mental or physical illnesses were excluded. The ages of participants ranged from 10 to 19 (mean = 15, SD = 2.13) years. The demographic descriptive statistics for study participants are further illustrated in Table 1.

**Table 1:** Demographic descriptive statistics.

Variables	*N* (%)	*𝜒^2^*	*p*
Gender		92.16^***^	<0.001
Male	172 (30.0%)		
Female	402 (70.0%)		
Grade		225.68^***^	<0.001
Primary school	26 (4.5%)		
Junior high school	307 (53.5%)		
Senior high school	241 (42.0%)		

*p < 0.05; **p < 0.01; ***p < 0.001.

### 2.2. Measures

The depressive symptoms of the participants were assessed using the HAMD-24 scale, which was developed by Hamilton in 1960 and is the most common scale used in the clinical assessment of depressive states (12). The Chinese version of the HAMD-24 has good reliability and validity, with factor coefficients of 0.83–1.00 (13). There are seven metrics in the HAMD-24 scale, which include anxiety, weight loss, cognitive disorder, diurnal variation, retardation, sleep disorder, and helplessness. Items were scored on a five-point scale from 0 to 4, and a higher total score indicates more severe depressive symptoms. Rating standards were as follows: severe depression (total score > 35), moderate depression (total score 20–35), mild depression (total score 8–20), and no depression (total score < 8). Each patient was examined jointly by two trained raters.

### 2.3. Statistical analysis

The dataset was analyzed using SPSS 26.0 (IBM, United States). For categorical variables such as demographic data, Chi-squared tests were used for analysis. The HAMD-24 scores of the patients were normally distributed and tested by an independent-sample t-test. One-way ANOVA was used to compare the difference between groups. All levels of significance were set at 0.05 (two-sided).

The LPA was conducted using Mplus software (version 8.0) with the aim of identifying potential classes associated with different genders. The optimal number of potential classes was determined based on a series of model fit indices, including AIC, BIC, ABIC, entropy, LMR-LRT, and BLRT (14). After identifying classes from the most optimal possible class models, each class is labeled based on a subjective interpretation of the data to facilitate future reference to these groups. ANOVA and Chi-square tests were used to measure the distribution of the categories identified by socio-demographic factors. Finally, further post-hoc analysis was performed using the Bonferroni correction method if necessary.

## 3. Results

### 3.1. Socio-demographic characteristics

Table 1 shows the socio-demographic characteristics and the Chi-squared test results of the participants. Our findings showed that the number of adolescents with depression who had visited the clinic was significantly greater among females than males (70.0% vs. 30.0%). In terms of school segments, middle school students were the largest group (53.5%), followed by high school students (42.0%) and upper elementary school students (4.5%).

### 3.2. Gender differences

Table 2 shows the portion of children with different levels of depressive symptoms as well as the documented differences between genders. Across age groups, female patients had significantly greater depressive levels than male patients. While mild and moderate depressive symptoms were most common among both genders, there were significant differences between males and females (*𝜒^2^* = 28.602^*^, *p* < 0.001), with mild depression predominating in male participants (57.6%) and moderate depression predominating in female participants (49.8%). As shown in Table 3, there are significant differences in all depressive symptoms except for weight loss in different genders.

**Table 2:** Gender difference in depression severity.

Variables	Male		Female		*𝜒^2^*	*p*
	*N*	*%*	*N*	*%*		
Mild	99	57.6%	137	34.1%	6.12^*^	0.013
Moderate	60	34.9%	200	49.8%	75.39^***^	<0.001
Severe	13	7.6%	65	16.2%	34.67^***^	<0.001

*p < 0.05; **p < 0.01; ***p < 0.001.

**Table 3:** Gender differences in all depressive symptoms.

Variables	Male		Female		*t*	*p*
	*M*	*SD*	*M*	*SD*		
Anxiety	5.016	0.285	5.545	0.218	−2.982^**^	0.003
Weight loss	0.395	0.087	0.376	0.066	−0.124	0.901
Cognitive disorder#	4.717	0.361	5.639	0.276	−3.736^***^	<0.001
Diurnal variation	0.623	0.077	0.871	0.059	−2.400^*^	0.017
Retardation	3.675	0.190	4.454	0.145	−4.774^***^	<0.001
Sleep disorder	2.066	0.226	3.124	0.173	−3.290^**^	0.001
Hopelessness#	3.258	0.273	4.045	0.209	−4.619^***^	<0.001
Total#	19.888	1.084	24.236	0.830	−4.995^***^	<0.001

### 3.3. Age differences

One-way ANOVA analysis showed significant differences among three age groups (primary school, junior high school, and senior high school) in the total scores calculated by the HAMD-24 scale [*F* (2,571) = 3.060*, *p* = 0.048]. The junior high school group had the most severe symptoms (mean = 24.599, SD = 0.537), followed by the senior high school group (mean = 23.336, SD = 0.606), and the primary school group (mean = 20.423, SD = 1.844). The post-hoc analysis demonstrated that depressive symptoms were significantly more severe in the junior high school group than in the primary school group (*t* = 1.920^*^, *p* = 0.030), while no differences were observed between the senior and junior high school groups (*t* = 0.809, *p* = 0.119).

### 3.4. LPA results

Considering significant gender differences in depressive symptoms, two separated LPA analyses were used to examine the pattern difference between the two gender groups. Table 4 displays the statistical fit indices of one- to four-class models. Based on pre-specified criteria of model selection, the two-class solution was chosen for the male group because it exhibited statistically significant *p*-values of LMR-LRT and BLRT, lower AIC, BIC, and ABIC values, and higher entropy than the 1-class, 3-class, and 4-class models. Similarly, the three-class solution was chosen for the female group since the LMR-LRT and BLRT values become nonsignificant after running the four-class model. Although the entropy value is slightly lower than the two- and four-class models, lower AIC, BIC, and ABIC values suggest that the three-class model may be the best fit.

**Table 4:** LPA result.

No. of classes	AIC	BIC	aBIC	Entropy	LMR-LRT	BLRT
Male						
1	3290.067	3334.132	3289.801			
2	**3032.379**	**3101.624**	**3031.961**	**0.916**	***p* < 0.0000**	***p* < 0.0000**
3	2980.259	3074.684	2979.689	0.930	*p* = 0.3865	*p* = 0.3966
4	2911.515	3031.12	2910.793	0.933	*p* = 0.1238	*p* = 0.1286
Female						
1	8065.156	8121.107	8076.683			
2	7464.351	7552.273	7482.465	0.821	*p* < 0.0000	*p* < 0.0000
3	**7294.749**	**7414.642**	**7319.45**	**0.820**	***p* = 0.0051**	***p* = 0.0055**
4	7111.162	7263.027	7142.449	0.872	*p* = 0.4358	*p* = 0.4406

Bold is for significant value.

**Figure 1:**
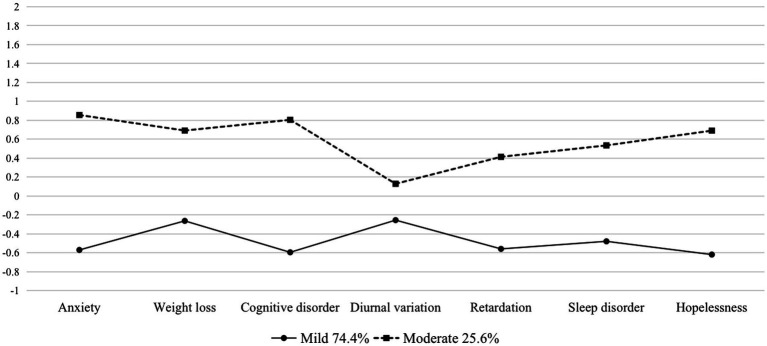
Latent classes for males (*N* = 172).

**Figure 2:**
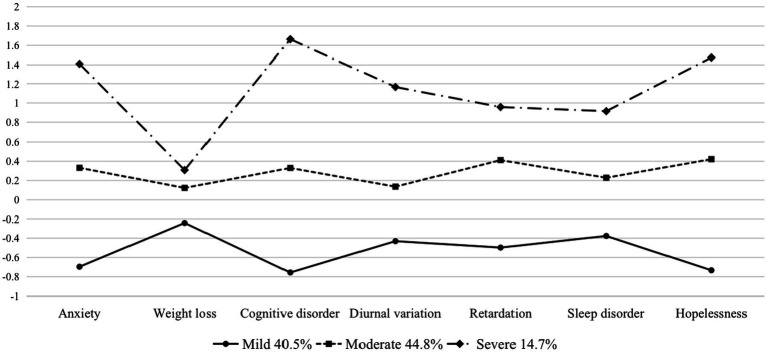
Latent classes for females (*N* = 402).

Figure 1 presents the two identified latent classes for male groups. The first class consisted of 74.4% of the male participants with relatively low levels of symptoms and was therefore labeled as “mild.” The other class comprised the remaining 25.6% of the male participants who had a moderate level of symptoms and were, hence, labeled as “moderate.” Figure 2 presents the three identified latent classes for female groups. The “mild” (40.5%) and “moderate” (44.8%) groups were quite similar compared to the male groups, while the third class, characterized by 14.7% of female adolescents who had a relatively higher level of symptoms, was labeled as “severe.”

Chi-square tests and ANOVA analyses showed that there were no significant differences in the distribution of socio-demographics across the classes. For the male group, grade and age were not significantly different across classes [*𝜒^2^* = 2.081, *p* = 0.353; *F* (2, 399) = 0.621, *p* = 0.538], and this finding was virtually identical to that in the female group [*𝜒^2^* = 6.941*, p* = 0.139; *F* (2, 399) = 1.319, *p* = 0.252]. However, when focusing on the perspective of depressive symptoms and total scores, there are significant differences across classes in both groups, as outlined in further detail in Table 5. The “mild” and “moderate” latent classes in both gender groups share the same characteristics in that their symptoms are relatively evenly distributed across the seven subscales. Notably, there is a distinctive group of females with severe depressive symptoms, characterized by more distinct scores in mental symptoms (i.e., cognitive disorder and hopelessness) rather than somatic symptoms (i.e., weight loss and sleep disorder).

**Table 5:** Difference across classes.

Variables	Male			Female			
	Class1	Class 2	*𝜒^2^/F & p value^ab^*	Class 1	Class 2	Class 3	*𝜒^2^/F & p value ^ab^*
	*N* = 128 (74.4%)	*N* = 44 (25.6%)		*N* = 163 (40.5%)	*N* = 180 (44.8%)	*N* = 59 (14.7%)	
Socio-demographic characteristics							
Grade *N* (%)							
Primary school	9 (7.0)	1 (2.3)	*𝜒^2^* = 2.08*, p* = 0.353	8 (4.9)	6 (3.3)	2 (3.4)	𝜒2 = 6.94, *p* = 0.139
Junior high school	67 (52.3)	21 (47.7)		84 (51.5)	94 (52.2)	41 (69.5)	
Senior high school	52 (40.6)	22 (50.0)		71 (43.6)	80 (44.4)	16 (27.1)	
Age Mean (SD)	15.45 (0.20)	15.91 (0.34)	*F* (1, 170) = 0.62, *p* = 0.538	15.36(0.16)	15.53 (0.15)	15.22 (0.27)	*F* (2, 399) =1.32, *p* = 0.252
HAMD-24 Mean (SD)							
Anxiety	4.16 (0.15)	7.61 (0.26)	*F* (1, 170) = 137.11, *p* < 0.001	3.73 (0.13)	6.39 (0.12)	9.03 (0.22)	*F* (2, 399) =247.61, *p* < 0.001
Weight loss	0.29 (0.06)	1.02 (0.10)	*F* (1, 170) = 38.77, *p* < 0.001	0.32 (0.06)	0.57 (0.05)	0.70 (0.09)	*F* (2, 399) =7.95, *p* < 0.001
Cognitive disorder	3.88 (0.17)	8.32 (0.29)	*F* (1, 170) = 175.83, *p* < 0.001	3.28 (0.14)	6.82 (0.14)	11.00 (0.24)	*F* (2, 399) =413.64, *p* < 0.001
Diurnal variation	0.64 (0.06)	0.91 (0.10)	*F* (1, 170) = 5.49, *p* = 0.020	0.52 (0.04)	0.90 (0.04)	1.63 (0.07)	*F* (2, 399) =90.22, *p* < 0.001
Retardation	3.34 (0.12)	5.00 (0.21)	*F* (1, 170) = 48.81, *p* < 0.001	3.44 (0.11)	4.96 (0.10)	5.83 (0.18)	*F* (2, 399) =84.37, *p* < 0.001
Sleep disorder	1.87 (0.15)	4.02 (0.25)	*F* (1, 170) = *53*.47, *p* < 0.001	2.07 (0.14)	3.31 (0.13)	4.64 (0.23)	*F* (2, 399) =52.96, *p* < 0.001
Hopelessness	2.67 (0.13)	5.80 (0.23)	*F* (1, 170) = 143.16, *p* < 0.001	2.36 (0.12)	5.12 (0.12)	7.71 (0.20)	*F* (2, 399) =292.76, *p* < 0.001
Total	17.07 (0.41)	32.82 (0.70)	*F* (1, 170) = 377.50, *p* < 0.001	15.86 (0.32)	28.16 (0.30)	41.09 (0.53)	*F* (2, 399) =921.74, *p* < 0.001

^a^𝜒^2^ test statistics are presented for categorical variables, F-test statistics for continuous variables. ^b^*p* < 0.05 indicated statistically significant between-class difference.

## 4. Discussion

Similar to previous findings, this study also found that the prevalence of depression in adolescent females was approximately twice that of adolescent males (15). As for the perspective of severity of depression symptoms, both inferential statistics and LPA analysis indicated that adolescent females are suffering more severe symptoms than their male counterparts.

According to the results of sub-scales, adolescent females showed a higher level of anxiety than boys. This is consistent with the findings from several previous studies showing that female adolescents reported more severe anxiety symptoms than male adolescents (16–18). Furthermore, cross-cultural studies have likewise shown that such gender differences are stable across studies conducted in multiple Eastern and Western countries, including China (19). In addition to difficult interpersonal relationships, higher levels of rumination in adolescent females (20–22) and higher levels of worry (22, 23) may further explain gender differences in adolescent anxiety problems (24).

The current study also showed that adolescent females suffer more from sleep disorders. Insomnia usually precedes adolescent depression and is highly comorbid with it (25–27). The studies on sleeping disorder also demonstrate differences in symptoms based on the genders of the individuals being studied, where insomnia remains equally common in boys and girls before adolescence (26, 27), while the prevalence of insomnia symptoms is much higher in females than in males after puberty (28). This difference may stem from several normal structural changes in adolescent sleep (e.g., the decrease in slow-wave N3 sleep), which begin earlier in girls (29). Additionally, pubertal development is associated with reduced melatonin levels (30), which may make it difficult for adolescents to obtain adequate and satisfactory sleep in the context of early morning school hours. Interestingly, as suggested by one recent study in adults, testosterone may be driving these gender differences in the evening while also offsetting the negative consequences of a lack of sleep that often accompanies delayed sleep times (31). Thus, it is possible that differences in testosterone production by adolescent males and females may also contribute to gender differences in sleep disorders. Additionally, higher heritability of insomnia in females (32) and behavioral and environmental influences may also contribute to gender differences in sleep disorders. For example, the use of electronic devices at bedtime has been found to suppress melatonin secretion and delay sleep onset. One study found that the correlation between electronic device use and shorter sleep duration was particularly significant in girls (33). Not only is the prevalence of insomnia higher among adolescent females, but also compared to adolescent males, insomnia is found to be a stronger predictor of depressive symptoms in girls (34), increasing the differences in depression between the two gender groups.

The current study also found that hopelessness was significantly more severe in adolescent females. Seligman’s theory of learned helplessness suggests that, in the face of repeated uncontrollable stressors, subjects will stop trying to master or overcome the stressor and eventually exhibit passive or apathetic behaviors (35). Research has shown that adolescents who feel hopeless are more likely to develop suicidal ideation and behaviors (36–39). More recent research has likewise shown that adolescents exposed to stressors they perceive as uncontrollable may exhibit passive or avoidant coping behaviors and experience despair (40). Negative attributional strategies contribute to the development of helplessness and symptoms of depression (41), and as mentioned earlier, females face more stress as well as differences in coping styles directly linked to gender (42), including negative or biased outlooks (43). As such, this cognitive vulnerability may be responsible for the higher level of females on the despair symptom subscale.

Although other symptom clusters (i.e., weight loss, cognitive disorder, diurnal variation, and retardation) do not benefit from the same degree of previous research where gender difference is concerned, there have been many previous studies that have attempted to explain gender differences in terms of the general depressive symptoms through individual variables. In recent years, researchers have begun to move toward explaining the onset of depression through comprehensive, interactional, and developmental models. First, females, in general, have lower social status or less social resources than males in most societies, and they experience certain traumas, especially sexual abuse, more often than their male counterparts (44). Females have also been shown to experience more chronic stressors such as poverty, harassment, lack of respect, and restricted choices (45). Second, even when women and men share the same stressors, there are gender differences in the self-concept of males and females (46), physiological responses (46, 47), and coping styles (45), which may lead to the fact that women are more likely to suffer from depression-related symptoms. Third, frequent experiences of stress and stress responses may interact differently between gender groups. Stressful experiences can sensitize the physiological and psychological systems to future stress, thus making individuals more likely to respond to depression. For example, reactions to stress are associated with impaired problem solving, in turn leading to a new accumulation of stress, which may likewise lead to more depressive symptomology (45). As the findings of this study show, this vicious circle leads females to suffer more from depression.

This study also found that both depression symptoms and prevalence were most severe in adolescents in the middle school group (with subjects 12–15 years of age) followed by the high school group, though the difference between the two was not significant. In a decade-long longitudinal study of over a 1,000 subjects in Dunedin, New Zealand, the period of rapidly increasing depression prevalence occurred between the ages of 15 and 18 years, with no significant increase before that time. Similar cross-national studies showed a comparable prevalence of psychiatric disorders, particularly depression, in the United States (48, 49). Based on these cross-national comparisons, findings regarding mental health in the Dunedin sample can be generalized to the United States and other industrial countries (6). Such findings suggest that the age distribution of adolescents with depression in China may be structurally different from that in Western countries, which may stem from differences in the education systems. While the United States promotes the value of individualism above collectivism and has a loosely managed classroom, China places great emphasis on learning cultural foundations from middle school onward, with strict classroom management and an emphasis on grades by both schools and parents. This means that Chinese students experience intense academic stress early on, which often hurts adolescents’ mental health (50). Therefore, it is reasonable that grades in school have a significantly stronger association with depressive symptoms among adolescents in China than those in the United States (51). This effect can be interactive, as intense academic stress can lead to depression, which in turn can affect academic performance, leading to light or even more severe depression.

The present study revealed some of the patterns and characteristics of Chinese adolescents with depression and provided hints for future research and clinical work. First, it is necessary to develop psychological or social interventions in schools and communities for adolescents who suffer from mental stress, especially those associated with study or school-related pressures. Second, it is still essential for Chinese society to boost equality for both genders by eliminating gender stereotypes, to make everyone free from extra hurt and free to express himself or herself. Last but not least, we are urging a culture-specific psychological treatment system for patients to fit our culture as well as the contemporary context.

## 5. Conclusion

This study highlights the gender differences in the prevalence and severity of depressive symptoms in Chinese adolescents. Our analyses showed that compared to males, females exhibited higher rates of depression and more severe depressive symptoms across age groups. In addition, the current study revealed an earlier onset of depression among adolescents in China compared to those in Western countries in previous studies. Gender differences have always been an important topic in China. Even in the present era, men and women face different dilemmas, which may be an important potential reason for gender differences in depression; promoting gender equality and developing gender-friendly interventions are extremely important for maintaining the overall mental health of adolescents in China. Finally, considering the cultural context, the authors advocate for more theoretical development and clinical practice on this issue in order to provide more localized and adaptive interventions for Chinese adolescents.

## Data availability statement

The raw data supporting the conclusions of this article will be made available by the authors, without undue reservation.

## Ethics statement

The studies involving human participants were reviewed and approved by Shanghai Pudong New Area Mental Health Ethics Committee. Written informed consent to participate in this study was provided by the participants’ legal guardian/next of kin.

## Author contributions

XF conceptualized and designed the study and critically reviewed the manuscript for important intellectual content. YS carried out the data analysis and drafted the initial manuscript. YZ revised the final manuscript. WS drafted the introduction and discussion parts of the initial manuscript. LC and JL took part in the revision of the manuscript. All authors contributed to the article and approved the submitted version.

## Funding

This work was supported by Medical discipline Construction Project of Pudong Health Committee of Shanghai (Grant No.: PWZzb2022-09), Science and Technology Development Fund of Shanghai Pudong New Area (Grant No.: PKJ2020-Y34), and Medical discipline Construction Project of Pudong Health Committee of Shanghai (Grant No.: PWYgy2021-02).

## Conflict of interest

The authors declare that the research was conducted in the absence of any commercial or financial relationships that could be construed as a potential conflict of interest.

## Publisher’s note

All claims expressed in this article are solely those of the authors and do not necessarily represent those of their affiliated organizations, or those of the publisher, the editors and the reviewers. Any product that may be evaluated in this article, or claim that may be made by its manufacturer, is not guaranteed or endorsed by the publisher.

## Abbreviations

WHO: World health organization
HAMD-24: Hamilton rating scale for depression-24
SD: Standard deviation
LPA: Latent profile analysis
AIC: Akaike information criterion
BIC: Bayesian information criterion
ABIC: Akaike’s Bayesian information criterion
LMR-LRT: Lo-Mendell-Rubin adjusted likelihood ratio test
BLRT: Bootstrap likelihood ratio test
